# Renal Function Mediates the Association Between Klotho and Congestive Heart Failure Among Middle-Aged and Older Individuals

**DOI:** 10.3389/fcvm.2022.802287

**Published:** 2022-04-18

**Authors:** Xu Zhu, Xinyi Lu, Ting Yin, Qingqing Zhu, Shi Shi, Iokfai Cheang, Xin Yue, Yuan Tang, Shengen Liao, Yanli Zhou, Haifeng Zhang, Xinli Li, Wenming Yao

**Affiliations:** ^1^Department of Cardiology, The First Affiliated Hospital of Nanjing Medical University, Jiangsu Province Hospital, Nanjing, China; ^2^Department of Cardiology, Affiliated Suzhou Hospital of Nanjing Medical University, Suzhou Municipal Hospital, Suzhou, China

**Keywords:** Klotho protein, congestive heart failure, cardiovascular disease, mediation analysis, NHANES

## Abstract

**Objective:**

Using a newly released National Health and Nutrition Examination Survey (NHANES) data of serum Klotho, this study aimed to explore the relationship between Klotho and specific cardiovascular diseases (CVD), as well as the mediation effect of renal function, among middle-aged and older individuals within the general population.

**Methods:**

This nationally representative cross-sectional study analyzed data from the 2007–2016 NHANES. A total of 13,765 participants, who aged 40 years or older, from the general population were examined. Klotho were divided into four groups based on median and interquartile range. The associations among Klotho (exposure), congestive heart failure (CHF; outcome), and renal function markers [estimated glomerular filtration rate (eGFR), blood urea nitrogen (BUN), uric acid (UA), and urine albumin-to-creatinine ratio (UACR); mediators] were investigated using mediation analysis.

**Results:**

In comparison to the lowest quartile, Klotho in the highest quartile was independently associated with the prevalence of CHF (OR 0.59; 95% CI 0.46–0.77, *p* for trend = 0.001), but not with other individual CVDs. Klotho had a significant direct effect on the prevalence of CHF (all *p* < 0.001), while eGFR, BUN, UA, and UACR partly mediated the indirect effect of Klotho on the prevalence of CHF (all *p* < 0.05), explaining 19.51, 6.98, 13.93, and 0.71% of the association between Klotho and CHF, respectively. Additionally, restricted cubic spline regression demonstrated a linear association and negative correlation between Klotho level and CHF.

**Conclusion:**

These findings suggest that Klotho is closely linked to CHF and renal function may be a key mediator of this association.

## Introduction

Soluble alpha-Klotho, abbreviated as Klotho, is a protein involved in anti-aging that is primarily located in the distal renal convoluted tubules, parathyroid gland, and brain (1). Klotho can exist in a soluble form (1), with reduced expression resulting in a condition comparable to that associated with human aging, including endothelial dysfunction, vascular calcification, and progressive atherosclerosis (2). Klotho appears to be capable of regulating intracellular calcium homeostasis and inhibiting reactive oxygen species, hence reducing myocardial hypertrophy, fibrosis, and cardiotoxicity (3). Additionally, studies have discovered that decreased soluble Klotho levels are related to left ventricular hypertrophy, myocardial fibrosis, and the prevalence of cardiovascular disease (4–8). These relationships are especially significant in patients with kidney illness, as Klotho is primarily produced within the kidney and its level lowers as renal function declines (9). Therefore, the underlying mechanism responsible for the direct effect between Klotho and cardiovascular disease (CVD) remains to be elucidated.

Cardiovascular disease is a serious complication of chronic kidney disease (CKD). CKD is associated with atherosclerosis, arrhythmia, heart failure, and cardiac fibrosis (10). CVD is still the main cause of morbidity and mortality in patients with CKD, even in the early stages (11). By 2040, it is estimated that CKD will become the fifth leading cause of mortality, with cardiovascular disease accounting for 20% of CKD deaths (12, 13). Studies have demonstrated a link between renal function markers and the prevalence or mortality of cardiovascular diseases (14–16).

Under normal physiological conditions, the kidney is the key regulator of serum Klotho levels (17). Klotho is closely associated with the pathogenesis of CKD. Recently, increasing evidence has shown that Klotho levels are lower in patients and animal models with renal insufficiency (18, 19), even though glomerular filtration rate (GFR) is only slightly decreased (20). In individuals with CKD, lower Klotho levels can be caused by a decrease in functional nephrons and inhibition of Klotho expression; however, Wnt/β-catenin activation is also regarded as a crucial component in Klotho reduction (21).

Few studies have investigated the correlation between Klotho and specific cardiovascular disease (CVD), and the potential links between Klotho, renal function, and heart failure remain unknown. Therefore, we explored the relationships between Klotho and specific CVD, and further quantified how the impact of Klotho on heart failure is mediated by renal function, based on a large and representative sample of American middle-aged and older individuals from the general population collected from the National Health and Nutrition Examination Survey (NHANES) from 2007 to 2016.

## Materials and Methods

### Study Population and Design

NHANES is a cross-sectional, nationally representative survey that aims to estimate the health and nutritional status of adults and children in the United States (22). The survey collects a representative sample of around 5,000 people per year. The survey contains demographic, socioeconomic, dietary, and health-related questions. The survey’s design, methods, and data are available to the public.^1^ The National Center for Health Statistics of the Centers for Disease Control and Prevention authorized the NHANES protocols, and all participants provide informed consent.

Our study investigated subsequent descriptive data from the five iterations of the continuous NHANES from 2007 to 2016. Among them, 36,823 participants were removed from the overall sample (50,588) because Klotho was not tested or because the participants were less than 40 years old. Removal of these participants resulted in a total of 13,765 subjects that were included for further analysis (Supplementary Figure 1).

### Covariates

We collected covariate data by questionnaires, physical examinations, and lab tests. The demographic characteristics of each subject, including age, sex, educational level, race, poverty income ratio (PIR), alcohol consumption, smoking status, hypertension history, and diabetes mellitus history were obtained during the family interview using a standardized questionnaire. Each subject’s height and weight were measured during body examination, and body mass index (BMI) was calculated. High-density lipoprotein cholesterol (HDL-C), TC (total cholesterol), Creatinine, blood urea nitrogen (BUN), uric acid (UA), and urine albumin-to-creatinine ratio (UACR) concentrations in blood samples were measured via laboratory analysis. The detail on blood collection and laboratory analysis process can be found at https://wwwn.cdc.gov/nchs/nhanes/continuousnhanes/labmethods.aspx?Cycle=2013-2014.

Educational levels were classified into three categories: below high school, high school, and above high school. Race and ethnicity were classified as follows: Mexican American, other Hispanic, non-Hispanic white, non-Hispanic black, and other race groups (including multi-racial). The family PIR was used to assess the ratio of family income to the poverty threshold. Poverty is characterized as a family PIR below one. A smoker was defined as someone who had smoked at least 100 cigarettes throughout the course of his or her lifetime. Alcohol users were identified as individuals who consumed at least 12 alcohol drinks in a single calendar year. Laboratory data was determined by standardized methods. BMI was calculated as weight in kilograms (kg) divided by height in meters squared (m^2^) (23). Sedentary time was assessed by interviewing participants. They were asked to report the number of minutes of sedentary activity during the day. Hypercholesterolemia was defined as total cholesterol over 200 mg/dL. The estimated GFR (eGFR) was computed using the Chronic Kidney Disease-Epidemiology Collaboration (CKD-EPI) equation (24). All of these variables are described on the NHANES website, which may be accessed at https://wwwn.cdc.gov/Nchs/Nhanes/continuousnhanes.

### Assessment of Klotho

In mobile examination centers (MECs), blood samples were obtained in the morning from individuals aged 1 and older who had fasted for at least 9 h. Workers received samples on dry ice and scrutinized each package. All samples were stored at -80°C until preset batches of samples were supplied to technicians for analysis on a regular basis. Klotho concentrations were determined using an enzyme-linked immunosorbent assay (ELISA; IBL international, Japan) on serum samples obtained from NHANES participants, aged 40–79, from 2007–2016, which were received and tested during 2019–2020 (25). The samples were examined twice, and the final value was calculated using the average of the two results. Each plate also contained two quality control samples (low and high concentration of Klotho). Samples with duplicate values differing by more than 10% were flagged for re-measurement. If the value of the quality control sample was not within two standard deviations of the known value, the entire plate was repeated. Klotho was detected at a lower limit of 6 pg/mL. The final values of all samples exceeded this limit. Details of the Klotho detection method can be found at: https://wwwn.cdc.gov/Nchs/Nhanes/2013-2014/SSKL_H.htm.

### Assessment of Cardiovascular Diseases Outcomes

In participants aged 40 years or older, CVD outcomes included self-reported physician diagnoses of congestive heart failure (CHF), coronary heart disease (CHD), angina, heart attack, or stroke. For the classification of specific CVD, participants were classed as having the disease (label = 1) if they replied “Yes” to the following cardiovascular symptoms and/or conditions: “Have you ever been told by a doctor that you had CHF, CHD, angina, a heart attack, or a stroke?” These were five distinct questions with identical phrasing.

### Statistical Analysis

We measured and analyzed Klotho concentrations and distributions. To verify the normal distribution of continuous variables, a Kolmogorov-Smirnov statistical test was performed. Klotho levels were log-transformed to normalize their distributions. The continuous variables of normal distribution were expressed as mean (standard deviation; SD) and compared with a one-way analysis of variance (ANOVA). Continuous variables with skewed distribution were described using median [interquartile range] and compared with a Kruskal-Wallis H test. Categorical or dichotomous variables were presented as absolute values (percentages) and compared with the chi-squared statistics.

We used logistic regression to calculate odds ratios (ORs) and 95% confidence intervals (CIs) to evaluate the prevalence of specific CVD linked with Klotho. Klotho was divided into quartiles, with the lowest quartile serving as the reference group. Model 1 was not adjusted by any covariate. Model 2 was adjusted for age, sex, education level, race, and poverty. Model 3 was the same as model 2 with additional adjustments for smoker, alcohol user, BMI, sedentary time, HDL-C, TC, diabetes mellitus, and hypertension. We conducted logistic analysis twice, using Klotho as continuous and categorical variables, respectively. To further investigate the dose-response curves between Klotho levels and CHF prevalence, restricted cubic splines with knots were used at the 10, 50, and 90th percentiles of Klotho level distribution.

To determine the extent to which the relationship between Klotho and CHF was mediated by renal function (eGFR, BUN, UA, and UACR), we used the R package (“mediation”) for causal mediation analyses to estimate the direct effect (DE), indirect effect (IE), and total effect (TE) (26). A mediator should be related to both the exposure and the outcome (27). In our study, Klotho, CHF, and renal function were independent variables, outcome variables, and mediating variables, respectively. This method is based on the framework of causal mediation analysis and is used to dissect the total effect of Klotho into a direct effect on the likelihood of developing CHF, and an indirect effect, via renal function (28). The mediation analysis was conducted using the PROCESS program with 5,000 bootstrap resamples and adjusted for the same covariates in model 3.

Finally, we conducted stratified analysis in multiple subgroups, including age (≤ 60 years versus > 60 years), sex (male versus female), obesity (BMI > 30 versus BMI ≤ 30), Hypercholesterolemia (yes versus no), diabetes (yes versus no), and hypertension (yes versus no), and examined the interaction between subgroups by a likelihood ratio test. IBM SPSS statistics (version 24.0) and R software (version 3.6.1) were used for all statistical analyses. Significance was set at *P* < 0.05 (two-sided).

## Results

### Population Characteristics of Participants According to the Quartile of Klotho Levels

In this study, 13,765 participants were included in the statistical analysis, with an average age of 57.7 (10.9) years and 6,667 males (48.4%). The overall prevalence of congestive heart failure, coronary heart disease, angina, heart attack, and stroke were 4.1% (*n* = 558), 5.1% (*n* = 699), 3.2% (*n* = 434), 5.4% (*n* = 743), and 4.5% (*n* = 624), respectively.

All participants were separated into four groups according to the quartile of baseline Klotho (pg/ml) (Q1 ≤ 654.65, 654.65 < Q2 ≤ 802.50, 802.50 < Q3 ≤ 993.35, Q4 > 993.35). A bar chart showing the skew distribution of Klotho levels and a bar chart depicting the normal distribution following log transformation can be found in Supplementary Figures 2A,B. Additionally, participants’ detailed baseline characteristics are summarized in Table 1. Participants in the highest Klotho category were more likely to be younger in age, female, more educated, non-smokers, and non-alcohol users. The highest proportion in each group were non-Hispanic whites, which decreased from the lowest to the highest quartile of Klotho levels. HDL-C and eGFR increased with higher Klotho levels, while BUN, UA, and UACR decreased. Additionally, those in the lowest quartile of Klotho levels were more likely to have diabetes, hypertension, CHF, CHD, heart attack, and stroke.

**TABLE 1:** Characteristics of the study population.

Variables	Klotho, pg/ml				*P*-value
	Q1, *n* = 3,442	Q2, *n* = 3,442	Q3, *n* = 3,440	Q4, *n* = 3,441	
Age, years	59.1 (11.1)	58.0 (10.8)	57.4 (10.7)	56.4 (10.6)	<0.001
Male, %	1,777 (51.6%)	1,780 (51.7%)	1,629 (47.4%)	1,481 (43.0%)	<0.001
**Education level, %**					0.001
Below high school	1,014 (29.5%)	969 (28.2%)	954 (27.7%)	961 (27.9%)	
High school	827 (24.0%)	747 (21.7%)	771 (22.4%)	704 (20.5%)	
Above high school	1,601 (46.5%)	1,726 (50.1%)	1,715 (49.9%)	1,776 (51.6%)	
**Race/ethnicity, %**					<0.001
Mexican American	558 (16.2%)	539 (15.7%)	560 (16.3%)	531 (15.4%)	
Other Hispanic	346 (10.1%)	384 (11.2%)	410 (11.9%)	437 (12.7%)	
Non-Hispanic White	1,555 (45.2%)	1,592 (46.3%)	1,504 (43.7%)	1,270 (36.9%)	
Non-Hispanic Black	698 (20.3%)	569 (16.5%)	585 (17.0%)	875 (25.4%)	
Other race	285 (8.3%)	358 (10.4%)	381 (11.1%)	328 (9.5%)	
Poverty, %	724 (21.0%)	681 (19.8%)	671 (19.5%)	715 (20.8%)	0.316
BMI, kg/m^2^	29.9 (6.4)	29.7 (6.6)	29.7 (6.7)	29.7 (7.0)	0.439
Smoker, %	1,863 (54.1%)	1,738 (50.5%)	1,610 (46.8%)	1,472 (42.8%)	<0.001
Alcohol user, %	2,566 (74.5%)	2,449 (71.2%)	2,389 (69.4%)	2,237 (65.0%)	<0.001
**Sedentary time, hours**					0.462
<3 h	933 (27.1%)	965 (28.0%)	961 (27.9%)	1003 (29.1%)	
3–6 h	1,243 (36.1%)	1,188 (34.5%)	1,184 (34.4%)	1,170 (34.0%)	
>6 h	1,266 (36.9%)	1,289 (37.4%)	1,295 (37.6%)	1,268 (36.8%)	
HDL-C, mmol/L	52.8 (17.0)	52.5 (16.5)	53.2 (16.7)	53.8 (16.8)	0.010
TC, mmol/L	198.9 (45.4)	199.1 (42.1)	198.9 (40.5)	198.5 (42.6)	0.925
UALB, ug/mL	8.5 [4.2, 21.3]	8.1 [4.1, 18.0]	7.8 [4.1, 16.0]	8.2 [4.1, 17.6]	<0.001
Creatinine, mg/dL	1.02 (0.76)	0.9 (0.4)	0.9 (0.4)	0.9 (0.4)	<0.001
eGFR, ml/min/1.73 m^2^	84.3 (24.3)	88.3 (21.5)	90.3 (20.4)	92.9 (20.4)	<0.001
BUN, mg/dL	15.3 (7.5)	14.4 (5.8)	13.9 (5.0)	13.3 (5.1)	<0.001
Uric acid, mg/dL	5.8 (1.5)	5.6 (1.5)	5.5 (1.4)	5.2 (1.4)	<0.001
UACR, mg/g	9.7 [5.1, 32.8]	8.7 [5.1, 25.3]	8.4 [4.7, 22.5]	8.5 [5.0, 22.0]	<0.001
Klotho, pg/ml	556.7 [489.7, 610.7]	727.5 [690.7, 763.5]	886.8 [841.6, 935.5]	1172.3 [1070.3, 1341.8]	<0.001
Diabetes mellitus, %	693 (20.1%)	582 (16.9%)	559 (16.3%)	642 (18.7%)	<0.001
Hypertension, %	1,771 (51.5%)	1,547 (44.9%)	1,530 (44.5%)	1,553 (45.1%)	<0.001
**Comorbid illness, %**					
CHF	205 (6.0%)	135 (3.9%)	117 (3.4%)	101 (2.9%)	<0.001
CHD	224 (6.5%)	173 (5.0%)	163 (4.7%)	139 (4.0%)	<0.001
Angina	121 (3.5%)	102 (3.0%)	104 (3.0%)	107 (3.1%)	0.553
Heart attack	238 (6.9%)	196 (5.7%)	159 (4.6%)	150 (4.4%)	<0.001
Stroke	196 (5.7%)	151 (4.4%)	144 (4.2%)	133 (3.9%)	0.002

*Continuous variables are expressed as mean ± SD or median [interquartile range] and categorical variables are expressed as number (%). Klotho was divided to four levels by quartile (Q1 ≤ 654.7; 654.7 < Q2 ≤ 802.5; 802.5 < Q3 ≤ 993.3; Q4 > 993.3). BMI, body mass index; HDL-C, high-density lipoprotein cholesterol; TC, total cholesterol; UALB, urinary albumin; eGFR, estimated glomerular filtration rate; BUN, blood urea nitrogen; UACR, urine albumin-to-creatinine ratio; CHF, congestive heart failure; CHD, coronary heart disease.*

### Multiple Logistic Regression Analysis Between Klotho and Specific Cardiovascular Diseases

The relationships between serum Klotho and prevalence of specific CVD were examined using multivariate logistic regression models. Univariate logistic analyses revealed significant correlations between Klotho levels and the prevalence of CHF, CHD, heart attack, and stroke (all *P* < 0.001; Tables 2, 3). After adjusting for models 2 and 3, Klotho levels were significantly associated with the prevalence of CHF (OR 0.64; 95% CI 0.54–0.77) and stroke (OR 0.84; 95% CI 0.71–1.00; Table 2). Furthermore, according to the interquartile range, Klotho levels were classified into quartiles (Q1, Q2, Q3, and Q4). After adjusting for multivariate models and using the lowest quartile of Klotho as the reference, the highest quartile of Klotho had a significant negative association with CHF (OR 0.59; 95% CI 0.46–0.77, *P* for trend = 0.001), but no correlation with other specific CVD (Table 3).

**TABLE 2:** Logistic regression results for relationship between Klotho (log2 transformation) and specific cardiovascular disease (CVD).

Individual CVDs	Model 1	Model 2	Model 3
	OR (95% CI)	OR (95% CI)	OR (95% CI)
Congestive heart failure	0.53 (0.44–0.63)^c^	0.61 (0.51–0.73)^c^	0.65 (0.54–0.78)^c^
Coronary heart disease	0.70 (0.60–0.82)^c^	0.87 (0.74–1.03)	0.92 (0.78–1.08)
Angina	0.95 (0.78–1.15)	1.10 (0.90–1.35)	1.18 (0.96–1.44)
Heart attack	0.69 (0.59–0.81)^c^	0.84 (0.72–0.99)^a^	0.89 (0.76–1.05)
Stroke	0.70 (0.60–0.83)^c^	0.80 (0.68–0.95)^a^	0.85 (0.72–1.00)^a^

*Model 1 was not adjusted by any covariate.*

*Model 2 was adjusted for age, sex, education level, race, and poverty.*

*Model 3 was adjusted as model 2 plus smoker, alcohol user, body mass index, sedentary time, high-density lipoprotein cholesterol, total cholesterol, urinary albumin, diabetes mellitus and hypertension.*

*OR, Odd ratio; CI, confidence interval. ^a^p < 0.05, ^b^p < 0.01 and ^c^p < 0.001.*

**TABLE 3:** Logistic regression results for relationship between Klotho and specific cardiovascular disease (CVD).

Specific CVD	Q1	Q2	Q3	Q4	*p-t*
	OR	OR (95% CI)	OR (95% CI)	OR (95% CI)	
**Congestive heart failure**					
Model1	1	0.65 (0.52–0.81)^c^	0.56 (0.44–0.70)^c^	0.48 (0.37–0.61)^c^	<0.001
Model2	1	0.70 (0.56–0.88)^b^	0.64 (0.51–0.81)^c^	0.57 (0.44–0.73)^c^	<0.001
Model3	1	0.75 (0.59–0.95)^a^	0.71 (0.55–0.90)^b^	0.60 (0.47–0.78)^c^	<0.001
**Coronary heart disease**					
Model1	1	0.76 (0.62–0.93)^b^	0.72 (0.58–0.88)^b^	0.61 (0.49–0.75)^c^	<0.001
Model2	1	0.82 (0.66–1.01)	0.84 (0.68–1.05)	0.80 (0.64–1.01)	0.149
Model3	1	0.88 (0.71–1.09)	0.92 (0.74–1.15)	0.85 (0.68–1.07)	0.492
**Angina**					
Model1	1	0.84 (0.64–1.10)	0.86 (0.66–1.12)	0.88 (0.68–1.15)	0.553
Model2	1	0.89 (0.68–1.17)	0.96 (0.73–1.25)	1.07 (0.82–1.40)	0.610
Model3	1	0.96 (0.73–1.27)	1.07 (0.81–1.41)	1.17 (0.89–1.54)	0.545
**Heart attack**					
Model1	1	0.81 (0.67–0.99)^a^	0.65 (0.53–0.80)^c^	0.61 (0.50–0.76)^c^	<0.001
Model2	1	0.88 (0.72–1.07)	0.76 (0.62–0.94)^a^	0.79 (0.64–0.98)^a^	0.047
Model3	1	0.94 (0.76–1.15)	0.83 (0.67–1.03)	0.84 (0.67–1.05)	0.269
**Stroke**					
Model1	1	0.76 (0.61–0.95)^a^	0.72 (0.58–0.90)^b^	0.67 (0.53–0.83)^c^	0.002
Model2	1	0.84 (0.67–1.05)	0.83 (0.67–1.04)	0.78 (0.62–0.98)^a^	0.152
Model3	1	0.90 (0.72–1.13)	0.90 (0.72–1.13)	0.83 (0.65–1.05)	0.450

*Model 1 was not adjusted by any covariate.*

*Model 2 was adjusted for age, sex, education level, race and poverty.*

*Model 3 was adjusted as model 2 plus smoker, alcohol user, body mass index, sedentary time, high-density lipoprotein cholesterol, total cholesterol, urinary albumin, diabetes mellitus and hypertension.*

*Klotho (pg/ml) was divided to four levels by quartile (Q1 ≤ 654.7; 654.7 < Q2 ≤ 802.5; 802.5 < Q3 ≤ 993.3; Q4 > 993.3).*

*OR, Odd ratio; CI, confidence interval; p-t: p for trend; ^a^p < 0.05, ^b^p < 0.01 and ^c^p < 0.001.*

**FIGURE 1:**
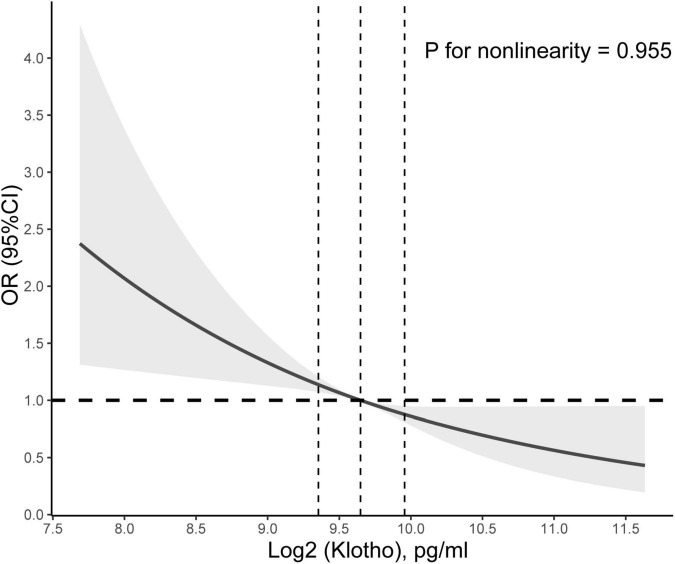
Association between Klotho level and congestive heart failure (CHF). Adjusted odds ratio of CHF from a restricted cubic spline logistic regression model with knots at the 10, 50, and 90th percentiles. Adjusted for age, sex, education level, race, poverty, smoker, alcohol user, body mass index, sedentary time, high-density lipoprotein cholesterol, total cholesterol, urinary albumin, diabetes mellitus, and hypertension. The solid and dashed lines represent the odds ratios and corresponding 95% confidence intervals. Dashed vertical lines are plotted at each quartile of Klotho level.

In addition, restricted cubic spline with a multivariate logistic regression model demonstrated that Klotho level had a linear association and a negative correlation with CHF (P for non-linearity = 0.754; Figure 1), whereas no relationship between Klotho level and coronary heart disease, angina, heart attack, or stroke was significant (Supplementary Figures 3A-D).

### Effect of the Mediators on the Relationship Between Klotho and Congestive Heart Failure

**FIGURE 2:**
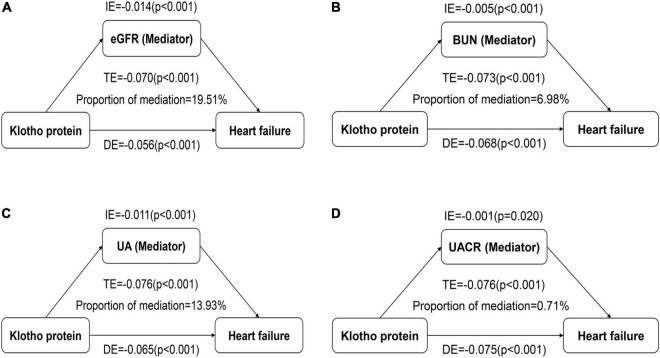
Effect of the renal function markers (mediators) on the relationship between Klotho (exposure) and congestive heart failure (CHF; outcome). **(A)** Mediation analysis for indirect effect of estimated glomerular filtration rate (eGFR); **(B)** Mediation analysis for indirect effect of blood urea nitrogen (BUN); **(C)** Mediation analysis for indirect effect of uric acid (UA); **(D)** Mediation analysis for indirect effect of urine albumin-to-creatinine ratio (UACR). DE, direct effect; IE, indirect effect; TE, total effect.

We conducted a mediation analysis to assess the extent to which renal function mediated the relationship between Klotho and CHF. After controlling for the model 3 described above, Klotho had a significant direct effect on the prevalence of CHF (all *p* < 0.001), and eGFR, BUN, UA, and UACR partly mediated the indirect effect of Klotho on the prevalence of CHF (all *p* < 0.05), with eGFR, BUN, UA, and UACR estimated to explain 19.51, 6.98, 13.93, and 0.71% of the association between Klotho and CHF, respectively (Figures 2A–D).

### Subgroup Analyses

Subgroup analysis was used to determine whether subgroups had different effects on the relationship between Klotho and CHF (Supplementary Table 1). After adjusting for confounding factors in model 3, and taking the bottom quartile of Klotho as the reference, the results showed no significant association between Klotho and CHF in some subgroups, including females, individuals with hypercholesterolemia, and non-hypertension participants. Additionally, there were no statistically significant interaction terms for any of the six variables considered (all *p* for interactions > 0.05).

## Discussion

In this cross-sectional study involving 13,765 middle-aged and older individuals in the general population of the United States, we investigated correlations between Klotho and specific CVD, and analyzed the mediation effects of renal function (eGFR, BUN, UA, and UACR) on these associations. Our findings show that serum Klotho is independently related to CHF, but displays no correlations with other CVDs, including CHD, angina, heart attack, and stroke. The prevalence of CHF is higher in middle-aged and older individuals with lower serum Klotho concentrations, and there was a linear association and a negative correlation between serum Klotho concentrations and CHF. Moreover, the relationships between Klotho and CHF are partially mediated by renal function, which could provide clues for the underlying mechanism of the positive effect of Klotho level on the prevalence of CHF.

Physiological homeostasis is challenged throughout life, and the ability to cope with such challenges decreases with age. Gender differences in life expectancy exist in humans (29). Extrinsic factors and/or intrinsic proximate mechanisms, such as hormone shifts, have been linked to sex-specific differences (29). Klotho is a human intrinsic factor associated to aging. Both sexes show a natural age-related decline in Klotho, but women have higher levels than men of the same age throughout their lives (30). In healthy men, smoking and psychological stress significantly increase Klotho levels (31). This increase in Klotho could be due to compensation for smoking’s detrimental consequences, such as systemic inflammation. Similarly, there is a possibility that Klotho is produced as a compensatory response and protects the heart during heart failure by acting as a suppressor of inflammation. For more educated, non-drinkers, life expectancy may be longer, which makes it easy to understand that these individuals may have higher levels of Klotho expression (31, 32).

Cardiovascular disease is the leading cause of mortality in the United States and around the world (33). Klotho protection is critical for the cardiovascular system to function properly. Klotho in its soluble form is acknowledged to have autocrine, endocrine, and paracrine hormone activities (4). Increasing evidence suggests that Klotho regulates intracellular Ca^2+^ homeostasis and inhibits reactive oxygen species levels to mitigate cardiac hypertrophy, fibrosis and cardiotoxicity (3, 34–36). In addition, the cardioprotective function of Klotho has also been attributed to its anti-apoptosis and pro-survival activities on endothelial cells and cardiomyocytes (37). Numerous studies have substantially enhanced our knowledge of Klotho’s antioxidative activity and its association with endothelial function (38). Klotho has been shown to regulate oxidative stress, fibrosis, and inflammation through suppression of insulin/insulin-like growth factor-1 and transforming growth factor-β1 signaling pathways (4, 38–40). Klotho may protect cells from oxidative stress-induced death (38). Klotho is a pleiotropic protein that has a number of beneficial effects on the endothelium. Endothelial nitric oxide synthase (eNOS) induces the generation of nitric oxide (NO), a recognized vasodilative factor that enhances endothelial cell activity (41). Klotho may inhibit adhesion molecule expression and is related to large increases in eNOS activity (39). In Klotho defective mice, research has indicated a reduction in NO production, endothelial dysfunction, and arteriosclerosis (2, 42). Klotho treatment may decrease lectin-like oxidized low-density lipoprotein receptor-1 (LOX-1) expression in endothelial cells, which plays a critical role in atherosclerosis development (43). Therefore, suppressing the LOX-1 pathway with Klotho alleviates inflammation and atherogenesis (43).

Although the precise mechanism responsible for the relationship between Klotho and CHF remains unclear, it has been demonstrated that there is a link between serum Klotho and traditional risk factors for CVD, or even the clinical history of CVD. Corsetti et al. (44) analyzed right atrial biopsy samples from twenty patients with CHD. Klotho was found to be expressed in myocardial tissue and was associated with the prevalence of CHD (44). The findings of our study were similar to those of a study conducted in Italy in 1,023 community-dwelling adults, which discovered that higher serum Klotho levels are independently associated with the prevalence of total CVD, which was defined as heart failure, CHD, stroke, and peripheral artery disease (6). Unfortunately, the authors did not perform separate analyses for specific CVDs. The present study, again a cross-sectional study, showed that in the general population, Klotho was only significantly negatively correlated with CHF, and has not with other specific CVDs. In addition, there are several longitudinal studies assessing the association of Klotho with prognosis (cardiovascular death and rehospitalization for heart failure) in patients with cardiovascular disease. The results of one study showed that low Klotho concentration is associated with an increased risk of cardiovascular death or heart failure hospitalization in patients with stable ischaemic heart disease (45). However, again in 969 stable coronary patients, no association of Klotho with poor patient prognosis was observed at a median follow-up of 5.39 years (46). Similar results were also observed in patients with CKD stages 2-4 and referred for coronary angiography (47, 48). To date, no studies have reported the relationship between Klotho and the prognosis of patients with heart failure. Thus, more studies may be needed to further confirm this in the future.

Klotho deficiency is associated with renal insufficiency (19). Klotho has been found in various studies to protect kidneys from loss of function (18). Meanwhile, acute kidney injury (AKI)/CKD are states of systemic Klotho deficiency, making Klotho a sensitive biomarker of impaired renal function (49). Klotho expression was dramatically decreased in patients with CKD following hemodialysis (20, 50). Klotho overexpression or administration has been shown in numerous preclinical models to have nephroprotective and cardioprotective effects (51–55). Klotho administration promotes kidney healing and limits renal fibrosis in mice following ischemia-reperfusion injury, hence slowing the progression of AKI to CKD (52, 56). Additionally, Klotho protects heart function and inhibits cardiac hypertrophy and fibrosis in patients with AKI (52). Given that CKD is related to the prevalence of CHF, the repletion of Klotho production may give renal protection and, as a result, may help to decrease the prevalence of CHF (57). Dysregulation of Klotho axis leads to higher cardiovascular risk resulting in increased cardiovascular morbidity and mortality rates of CKD patients (58). In our study, renal function was found to significantly mediate the association between Klotho and CHF, which may signify Klotho playing important roles in the pathogenesis of CHF. Our findings, however, should be interpreted cautiously, as while the mediation analysis is undoubtedly useful, the results of cross-sectional design are just relevant and do not imply causation (59).

This is the first study to examine the relationship between Klotho and CHF in the general population of middle-aged and older adults. The present study’s strengths include a large sample size and a substantial amount of data on conventional cardiovascular risk factors. We not only investigated the association between Klotho and CHF, but also putative mechanisms using mediation analysis. There are several important limitations to this study that should be taken into account. To begin, the cross-sectional design of the study precluded us from establishing a causal association between Klotho levels and CHF, which needs to be further validated in prospective cohort studies. Second, as with any epidemiological investigation, unmeasured confounding variables (e.g., LDL-C) may impact the association between Klotho and CHF. In addition, the outcome variables were based on self-reported CVD history and may not be completely accurate. However, studies have shown that self-reported outcomes in NHANES are a valid tool for assessing prevalence (60). Finally, because the current study focused on middle-aged and older individuals in the general population of the United States, the results may not be generalizable to people of all ages, health conditions, or ethnic origins.

## Conclusion

In conclusion, this is the first study to demonstrate that, in the general population, middle-aged and older adults with higher serum Klotho levels have a lower prevalence of CHF. Renal function is an important mediator in the observed relationship between Klotho and CHF. Prospective and mechanistic studies are necessary to validate and expand these findings, as well as to explore whether serum Klotho concentrations are predictive of incidence of CHF.

## Data Availability Statement

The datasets presented in this study can be found in online repositories. The names of the repository/repositories and accession number(s) can be found in the article/Supplementary Material.

## Ethics Statement

The studies involving human participants were reviewed and approved by the NCHS Ethic Review Board. The patients/participants provided their written informed consent to participate in this study. Written informed consent was obtained from the individual(s) for the publication of any potentially identifiable images or data included in this article.

## Author Contributions

XZ: conceptualization, methodology, software, formal analysis, writing–original draft, and visualization. XyL: conceptualization, methodology, writing–original draft, and supervision. TY: formal analysis and data curation. QZ: formal analysis and methodology. SS and SL: data curation and writing–review and editing. IC and YT: writing–review and editing. XY and YZ: project administration and writing–review and editing. HZ: data curation. XlL: conceptualization, methodology, writing–review and editing, and supervision. WY: conceptualization, methodology, project administration, writing–review and editing, and supervision. All authors contributed to the article and approved the submitted version.

## Conflict of Interest

The authors declare that the research was conducted in the absence of any commercial or financial relationships that could be construed as a potential conflict of interest.

## Publisher’s Note

All claims expressed in this article are solely those of the authors and do not necessarily represent those of their affiliated organizations, or those of the publisher, the editors and the reviewers. Any product that may be evaluated in this article, or claim that may be made by its manufacturer, is not guaranteed or endorsed by the publisher.

## Footnotes

1
https://www.cdc.gov/nchs/nhanes/about_nhanes.htm

## Abbreviations

NHANES: National Health and Nutrition Examination Survey
CVD: cardiovascular diseases
CHF: congestive heart failure
eGFR: estimated glomerular filtration rate
BUN: blood urea nitrogen
UA: uric acid
UACR: urine albumin-to-creatinine ratio
CKD: chronic kidney disease
PIR: poverty income ratio
BMI: body mass index
HDL-C: high-density lipoprotein cholesterol
TC: total cholesterol
CHD: coronary heart disease
ORs: odds ratios
CIs: confidence intervals
DE: direct effect
IE: indirect effect
TE: total effect
AKI: acute kidney injury.
