# Supplementary material for: Renal Function Mediates the Association Between Klotho and Congestive Heart Failure Among Middle-Aged and Older Individuals
Source: Front Cardiovasc Med. 2022 Apr 18;9:802287. doi: 10.3389/fcvm.2022.802287 (PMC9058082; doi:10.3389/fcvm.2022.802287)
Supplement: Supplementary file 1 [file Data_Sheet_1.docx]

**
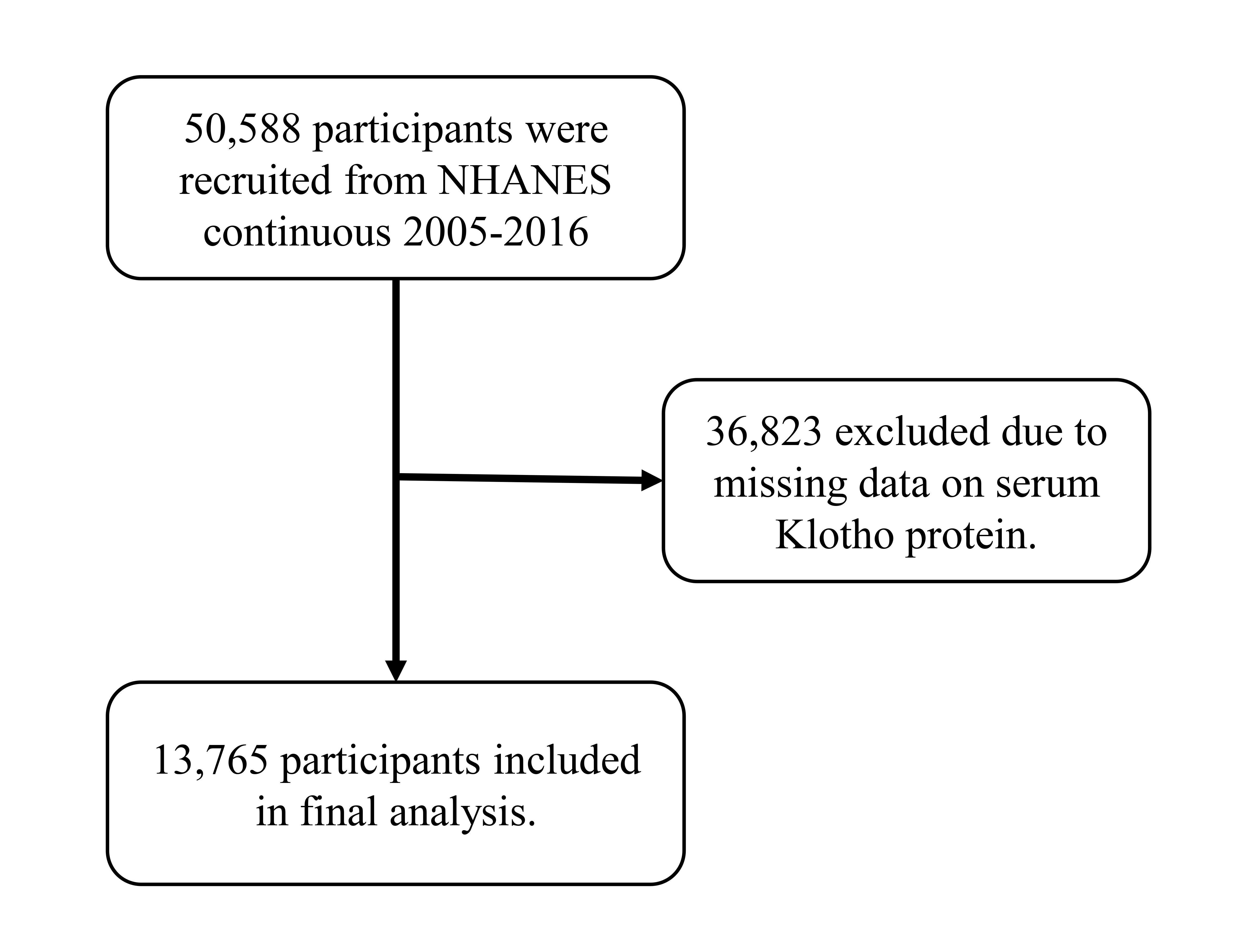
**

**Supplemental Figure 1**. Eligible participants in the evaluation of the influence between Klotho and cardiovascular disease (CVD) in middle-aged and older individuals.


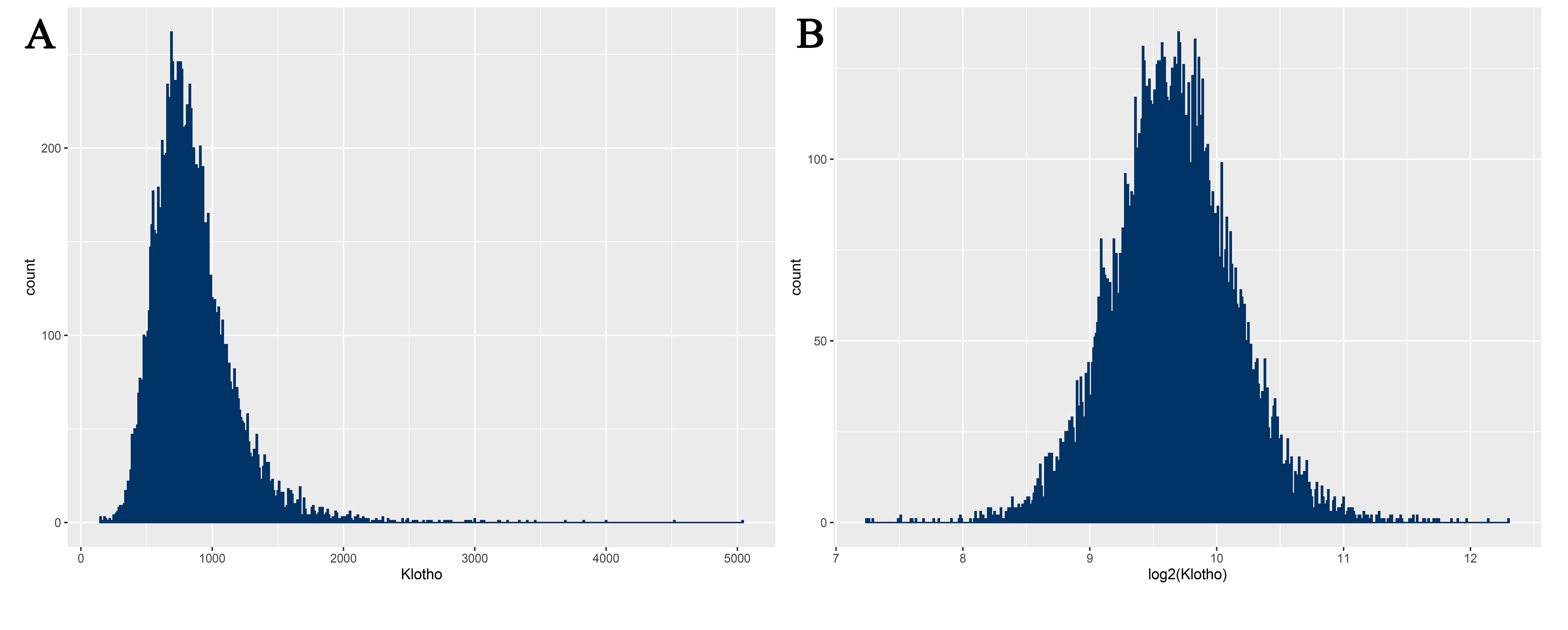


**Supplemental Figure 2**. Distributions of serum Klotho concentrations (A); Log-transformed distributions of serum Klotho concentrations (B).


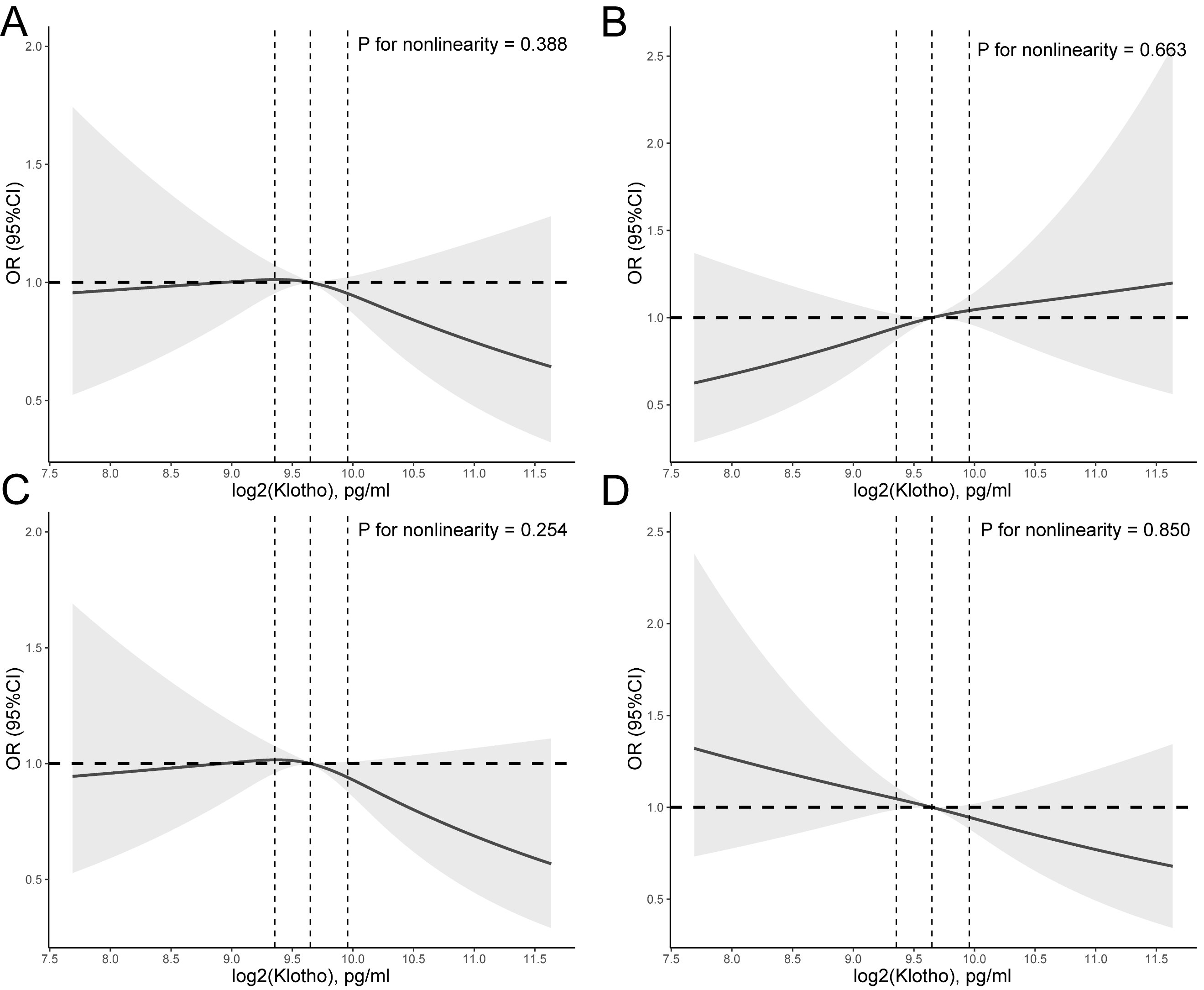


**Supplemental Figure 3**. Association between Klotho level and (A) coronary heart disease, (B) angina, (C) heart attack, and (D) stroke. Adjusted odds ratio of CHF from a restricted cubic spline logistic regression model with knots at the 10th, 50th, and 90th percentiles. Adjusted for age, sex, education level, race, poverty, smoker, alcohol user, body mass index, high-density lipoprotein cholesterol, total cholesterol, urinary albumin, diabetes mellitus and hypertension. The solid and dashed lines represent the odds ratios and corresponding 95% confidence intervals. Dashed vertical lines are plotted at each quartile of Klotho protein level.

**Supplementary Table S1.** Subgroups analysis for the associations of Klotho and congestive heart failure.

| Subgroups | Q1 | Q2 | Q3 | Q4 | *p-t* | *p-int* |
| --- | --- | --- | --- | --- | --- | --- |
|  | OR | OR (95% CI) | OR (95% CI) | OR (95% CI) |  |  |
| Age |  |  |  |  |  |  |
| ≤60 years | 1 | 0.55(0.36-0.85) ^b^ | 0.61(0.40-0.94) ^a^ | 0.44(0.28-0.68) ^c^ | 0.001 | 0.261 |
| > 60 years | 1 | 0.84(0.63-1.10) | 0.73(0.54-0.98) ^a^ | 0.68(0.50-0.93) ^a^ | 0.054 |  |
| Sex |  |  |  |  |  |  |
| Male | 1 | 0.71(0.53-0.95) ^a^ | 0.58(0.42-0.81) ^b^ | 0.51(0.36-0.72) ^c^ | <0.001 | 0.356 |
| Female | 1 | 0.80(0.55-1.17) | 0.86(0.60-1.25) | 0.72(0.49-1.05) | 0.372 |  |
| Obesity |  |  |  |  |  |  |
| BMI>30 | 1 | 0.81(0.60-1.10) | 0.81(0.59-1.10) | 0.54(0.38-0.76) ^c^ | 0.006 | 0.180 |
| BMI≤30 | 1 | 0.68(0.47-0.98) ^a^ | 0.55(0.37-0.82) ^b^ | 0.67(0.45-0.98) ^a^ | 0.015 |  |
| Hypercholesterolemia | | | | | | |
| Yes | 1 | 1.03(0.67-1.57) | 0.73(0.46-1.16) | 0.70(0.43-1.12) | 0.249 | 0.376 |
| No | 1 | 0.64(0.49-0.85) ^b^ | 0.67(0.50-0.89) ^b^ | 0.57(0.42-0.76) ^c^ | <0.001 |  |
| Diabetes |  |  |  |  |  |  |
| Yes | 1 | 0.61(0.42-0.89) ^b^ | 0.58(0.39-0.86) ^b^ | 0.64(0.44-0.92) ^a^ | 0.008 | 0.219 |
| No | 1 | 0.83(0.62-1.12) | 0.78(0.56-1.03) | 0.53(0.37-0.75) ^c^ | 0.005 |  |
| Hypertension |  |  |  |  |  |  |
| Yes | 1 | 0.77(0.59-0.99) ^a^ | 0.64(0.49-0.84) ^b^ | 0.59(0.45-0.78) ^c^ | 0.001 | 0.458 |
| No | 1 | 0.63(0.36-1.11) | 0.92(0.54-1.57) | 0.58(0.31-1.07) | 0.196 |  |

Analyses was adjusted for age, sex, education level, race, poverty, smoker, alcohol user, body mass index, high-density lipoprotein cholesterol, total cholesterol, diabetes mellitus and hypertension.

Klotho (pg/ml) was divided to four levels by quartile (Q1 ≤ 654.7; 654.7 < Q2 ≤ 802.5; 802.5 < Q3≤ 993.3; Q4 > 993.3).

Abbreviations: OR, Odd ratio; CI, confidence interval; *p-t, p* for trend; *p-int*, *p* for interaction; ^a^ *p* < 0.05, ^b^ *p* < 0.01 and ^c^ *p* < 0.001.
